# Fouling Development in A/O-MBR under Low Organic Loading Condition and Identification of Key Bacteria for Biofilm Formations

**DOI:** 10.1038/s41598-018-29821-9

**Published:** 2018-07-30

**Authors:** Yuya Takimoto, Masashi Hatamoto, Takaya Ishida, Takahiro Watari, Takashi Yamaguchi

**Affiliations:** 1 Department of Science of Technology Innovation, Nagaoka University of Technology, 1603-1 Kamitomioka, Nagaoka, Niigata, 940-2188 Japan; 2 Department of Civil and Environmental Engineering, Nagaoka University of Technology, 1603-1 Kamitomioka, Nagaoka, Niigata, 940-2188 Japan; 3 Top Runner Incubation Center for Academia-Industry Fusion, Nagaoka University of Technology, 1603-1 Kamitomioka, Nagaoka, Niigata, 940-2188 Japan

## Abstract

Membrane fouling in membrane bioreactors (MBR) remains a major issue and knowledge of microbes associated with biofilm formation might facilitate the control of this phenomenon, Thus, an anoxic/oxic membrane bioreactor (A/O-MBR) was operated under an extremely low organic loading rate (0.002 kg-COD·m^−3^·day^−1^) to induce membrane fouling and the major biofilm-forming bacteria were identified. After operation under extremely low organic loading condition, the reactor showed accumulation of total nitrogen and phosphorus along with biofilm development on the membrane surface. Thus, membrane fouling induced by microbial cell lysis was considered to have occurred. Although no major changes were observed in the microbial community structure of the activated sludge in the MBR before and after membrane fouling, uncultured bacteria were specifically increased in the biofilm. Therefore, bacteria belonging to candidate phyla including TM6, OD1 and Gammaproteobacteria could be important biofilm-forming bacteria.

## Introduction

Large-scale membrane bioreactors (MBRs) have been recently developed for wastewater treatment and their capacity has been increased^1^. MBR can achieve high removal efficiency for nutrients and complete removal of suspended solids from treated water because of a combined system involving activated sludge with membrane filtration. In addition, the MBR has the potential to simplify and reduce the footprint of a wastewater treatment system. However, membrane fouling remains a major issue in MBRs; it causes membrane clogging and decreases permeation flux. Membrane fouling has been divided into two classes: reversible and irreversible fouling. The latter, called biofouling, is caused by microbial products derived from bacterial metabolism and lysis^2^. Microbial products such as extracellular polymer substances (EPS) and soluble microbial products (SMPs) induce mature biofilm formation, causing serious fouling associated with high membrane resistance^3,4^.

To date, bacteria related to biofilm formation have been determined in various MBRs treating several kinds of wastewater. The relationship between fouling development and bacterial species that show high productivity of foulants such as EPS, SMPs, and auto-inducers, has been studied and reported previously^5,6^. High bacterial relative abundance, high microbial community diversity, and high productivity of foulants probably has an important role in biofilm formation^7,8^. Further, the attachment and growth of pioneer bacteria belonging to Betaproteobacteria and Gammaproteobacteria on the membrane surface plays an important role in biofilm formation and might cause severe fouling^9,10^. Thus, characterization of fouling-related bacteria is important for the optimization of MBR operational conditions and fouling control. However, reports on bacteria related to biofilm formation detected on the fouled membrane surface in MBRs treating municipal wastewater are limited^9,11,12^. In addition, the existence of common biofilm-forming bacteria among various MBRs is still unclear.

Although various fouling control techniques have been reported, no anti-biofouling method has not been widely accepted yet, because the wastewater and operational conditions differ in each MBR^13–15^. Moreover, since reactor operation and fouling control are usually based on rules of thumb in each MBR plant, the mechanisms involved in bio-fouling and biofilm formation are also unclear. Considering the reactor parameters, many studies have focused on the EPS and SMP derived major microbes in the fouled MBR, and these components were found to increase under high organic loading rates or low temperature conditions^16,17^. Membrane fouling was also found to be caused by EPS production in long term starvation conditions^18^. Thus, considering the positive correlation between membrane fouling and microbial lysis occurring under starvation conditions, microbial lysis seems to be an important factor in the development of membrane fouling in the MBR.

The present study aimed to confirm a low organic loading rate condition induce membrane fouling and to estimate the biofilm-forming bacteria in an operating anoxic/oxic (A/O) -MBR treating actual municipal wastewater under the condition. Moreover, to elucidate the common biofilm-forming bacteria, the microbial community was compared to that in naturally induced biofouling in an A/O-MBR under the normal operational conditions. The similarity in bacterial types identified in two fouled reactors operated under different conditions was determined. The present study provides a new perspective on biofilm-forming bacteria in a biofilm from a fouled membrane surface.

## Methods

### A/O-MBR operational condition

**Figure 1:**
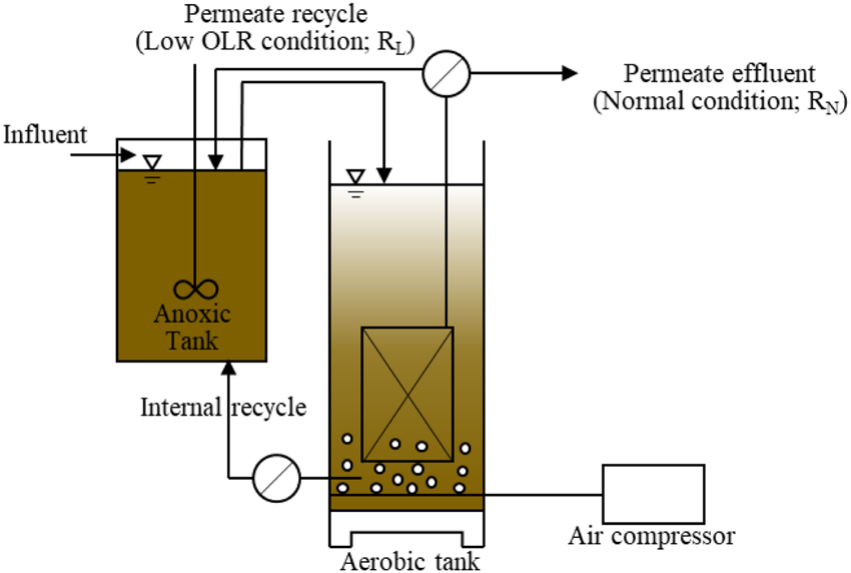
Schematic diagram of the A/O-MBR used in this study. A permeate effluent was recycled to the anoxic tank under the low OLR condition (R_L_).

Two lab-scale A/O-MBR systems designated R_L_ and R_N_, consisting of a 6 L anoxic tank and a 6 L aerobic tank, were used for the experiment (Fig. 1). The membrane module with 0.11 m^2^ filtration area and a chlorinated polyvinyl chloride flat sheet with 0.20 µm mean pore size (KUBOTA Co., Ltd., Japan) were submerged in the aerobic tank. Aeration was supplied by a diffuser at the bottom of the reactor. Anoxic and aerobic internal recycling was conducted to remove the phosphate and nitrogen. Municipal sewage after sedimentation was used as an influent into the anoxic tank. Table 1 shows the characteristics of the municipal sewage.

**Table 1:** Characteristics of the wastewater used in this study.

Parameters	Units	Average ± SD (N = 22)
Temperature	°C	16.1 ± 2.9
pH	—	6.77 ± 0.17
Dissolved COD	mg/L	156 ± 54
NH^4+^	mg-N/L	24.1 ± 5.2
NO^3−^	mg-N/L	0.13 ± 0.05
TN	mg/L	27.9 ± 7.4
TP	mg-P/L	2.4 ± 0.6

The hydraulic retention time (HRT) of the reactors was 8.0 h with a solid retention time (SRT) of 60 d. Each reactor was operated under the following conditions: A membrane suction cycle of 9 min on and 1 min off was adopted and an average membrane operating flux of 11.8 L·m^−2^·h^−1^ (LMH) with an aeration rate of 5.0 L/min was set. Conventional activated sludge (AS) taken from a sewage treatment facility, was seeded and the initial mixed liquor suspended solids (MLSS) concentration was approximately 4300 mg/L in each MBR. Both reactors were operated under the standard conditions of 0.42 kg-COD·m^−3^·day^−1^ until the reactor showed a stable performance. To induce membrane fouling, the permeate effluent of the R_L_ reactor was used to recycle into the anoxic tank to generate a low organic lading rate (OLR) starvation condition (0.002 kg-COD·m^−3^·day^−1^). To compensate the 200 ml of sampling of AS from the R_L_ reactor every day, 200 ml of sewage was fed as an influent, accounting for 0.002 kg-COD·m^−3^·day^−1^. On the other hand, the R_N_ reactor was continued to operate under standard conditions (0.42 kg-COD·m^−3^·day^−1^).

### Analytical methods

Temperature, pH, and dissolved oxygen (DO) of the AS in the aerobic tank and the oxidation-reduction potential (ORP) of AS in the anoxic tank were measured on-site using a portable pH, DO meter (DM-32P, TOA DKK, Japan), and ORP meter (HM-31P, TOA DKK, Japan), respectively. The permeate flow rate (30 minutes) was also measured on-site using a measuring cylinder. The transmembrane pressure (TMP) of each reactor was measured using a pressure transducer (ZSE50F, SMC, Japan) located in the permeate line. Dissolved COD, MLSS, ammonium, nitrite, nitrate, total nitrogen (TN) and total phosphate (TP) of samples were measured. Dissolved COD and TN were measured using water-quality analyzer (DR2800, Hach, USA). Ammonium, nitrite and nitrate concentrations were measured by HPLC (LC-20ADsp, SHIMADZU Co., Ltd. Japan). All samples were filtered using 0.2 µm filter paper.

### Biofilm sampling

After development of membrane fouling, the fouled membrane was taken from the aerobic tank and the membrane surface was rinsed with distilled water to remove the activated sludge attached to the membrane. The loosely bonded sludge cake on the fouled membrane surface was softly exfoliated and sampled as a membrane sludge (MS) sample using a thin plastic plate. Finally, the tightly bonded biofilm on the fouled membrane surface was sampled as a biofilm (BF) sample using a spatula. The samples were stored at −20 °C until DNA extraction.

### 16S rRNA genes analysis

The AS in the aerobic tank and the MS and BF on the membrane surface were used for microbial analysis. Genomic DNA from each sample was extracted using the FastDNA Spin Kit for Soil (MP Biomedicals, Santa Ana, CA). A forward universal bacterial primer Univ515F (5′-GTGCCAGCMGCCGCGGTAA-3′) and a reverse universal primer Univ806R (5′-GGACTACHVGGGTWTCTAAT-3′) were used in this study to amplify the bacterial 16S rRNA genes. PCR was performed using the following conditions: one cycle of 94 °C for 3 min, 25 cycles of 94 °C for 45 s, 50 °C for 60 s and 72 °C for 90 s, and a final cycle 72 °C for 10 min. The PCR products were purified using QIAquick PCR Purification Kit (QIAGEN, Germany), and 16S rRNA genes sequencing was performed as described by Caporaso *et al*.^19^. DNA was sequenced using the MiSeq Reagent Kit v2 and the MiSeq System (Illumina Inc., San Diego, CA).

### Data analysis

All data were analyzed using the QIIME software (version 1.9.1)^20^. Operational taxonomic units (OTUs) were selected at 97% identity using UCLUST. Taxonomic classification was assigned using BLAST based on the Greengenes database ver. 13_8. The relative species of predominant OTUs were searched using BLAST in the NCBI database (http://blast.ncbi.nlm.nih.gov/Blast.cgi). To compare the metabolisms and functional enzymes between the AS and BF samples, the Phylogenetic Investigation of Communities by Reconstruction of Unobserved States (PICRUSTs) based on the KEGG database was used^21^. A principal component analysis (PCA) plot with significant mean proportion differences for virginal datasets was created using Sequence Tag-based Analysis of Microbial Population dynamics (STAMP) software. The raw sequence data obtained in this study were deposited in the sequence read archive in the DDBJ database under the accession numbers DRA006840.

## Results and Discussion

### Fouling development and reactor performance

Both reactors were operated for about 6 months under standard conditions. After 6 months of operation, the membrane modules in the reactor were physically washed with ultra-pure water using sponges. Then, both reactors were used for the experimental study. In this study, the first day was defined as after about 3 weeks from the membrane wash. The R_L_ reactors showed the following performance after being operated at 3 weeks from membrane cleaning under standard conditions: TMP (8 kPa), flux (0.28 m/day), MLSS concentration (10195 mg/L), COD removal rate (82%), and TN removal rate (64%). On the other hand, the R_N_ reactor showed the following performance: TMP (6.2 kPa), flux (0.27 m/day), MLSS concentration (10280 mg/L), COD removal rate (83%), and TN removal rate (68%). After each MBR achieved a stable operational condition (upon operation at 3 weeks after washing), the R_L_ reactor was started to operate under the low OLR condition in order to induce membrane fouling development caused by microbial lysis. The R_N_ reactor was continued to operate under stable condition.

**Figure 2:**
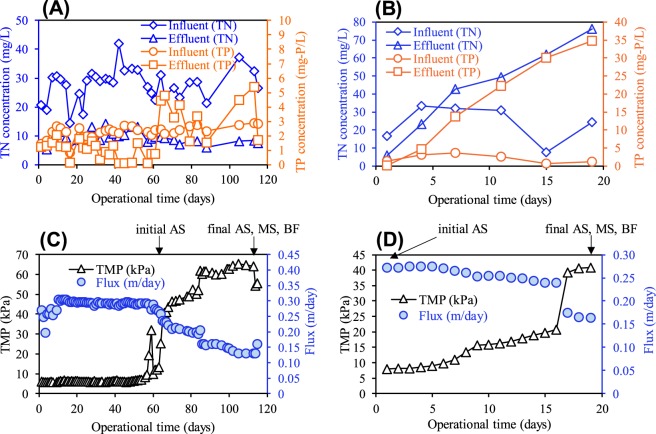
Performances of each reactor under different conditions. (**A**) and (**B**) shows the performance of TN and TP removal in the R_N_ reactor and R_L_ reactor, respectively. (**C**) and (**D**) shows the TMP and flux profiles during each operational condition after a stable operational term in the R_N_ reactor (**C**) and the R_L_ reactor (**D**). Arrows indicate the sampling points for microbial analysis and the sample name.

The performance of both MBRs under different conditions is shown in Fig. 2. Both MBRs in the initial phase reached approximately 80% dissolved COD removal, (data not shown). The removal efficiency of dissolved COD in the R_L_ and R_N_ reactor was stable until the final phase. However, the TN removal ratio of the R_L_ reactor began to deteriorate soon after initiating the low OLR operation (Fig. 2A,B). Although the TN and TP in the R_N_ reactor was stable until the final phase, their concentrations continued to increase during the operational term for the R_L_ reactor. A/O-MBR has high removal efficiency for nitrogen and phosphorus to possess the phosphorus accumulating organisms (PAOs) and denitrifying bacteria^22^. In this study, the removal efficiency for nitrogen and phosphorus was decreased in the R_L_ reactor. The average TN and TP concentrations in the influent were 27.9 ± 7.4 mg-N/L and 2.4 ± 0.6mg-P/L, respectively. Thus, the amount of nitrogen and phosphorus that flowed into the R_L_ reactor in a day was calculated only 5.6 mg-N/day and 0.5 mg-P/day on an average, respectively. However, the increasing rate of nutrient concentration far exceeded the amount of that in only the influent sewage of the R_L_ reactor. Therefore, the accumulated TN and TP were considered from the retained sludge in the R_L_ reactor. These results also implied that nucleic acids and microbial products derived from microbial lysis induced by low OLR conditions were released in the R_L_ reactor. Accordingly, the MLSS of the R_L_ reactor was decreased to 7566 mg/L at the final phase from 10195 mg/L at the initial phase (Fig. S1). The degradation of MLSS in the R_L_ reactor also suggested that microbial lysis occurred in the R_L_ reactor. On the other hand, in the R_N_ reactor, the TP was temporary accumulated and the MLSS concentration was drastically decreased from 59 days to 66 days. This result might suggest that microbial lysis also occurred in the R_N_ reactor as the TMP jump was observed.

The progression of fouling in each reactor was evaluated by monitoring the increase in TMP and the decrease of flux (Fig. 2C,D). In the R_N_ reactor, stable operation was continued for 2 months, and a drastic increase in TMP was observed at 64 days and flux was decreased from 0.27 m/day to 0.16 m/day with the TMP reaching 60 kPa after 86 days of operation. In contrast, a sudden increase of TMP to 40 kPa and decrease of flux of 0.28 m/day to 0.17 m/day was confirmed after 17 days after the low OLR condition was initiated in the R_L_ reactor. These results show that membrane fouling was developed under extremely the low organic loading rate condition (OLR: 0.002 kg-COD·m^−3^·day^−1^) of the R_L_ reactor. In the previous study, although higher fouling development at a high organic loading rate was reported^23^, induction of rapid and severe fouling development was confirmed at a low organic loading rate condition in this study.

The development of fouling behavior has been described as occurring in three or two stages^8,24^, and the changes in TMP in this study were also divided into three stages (Fig. 2C,D). In the R_L_ reactor, the stage from the first day to the 8th day was considered as initial fouling, or the first step of fouling. The second step was from the 9th day to the 16th day, and the third step was from the 17th day to the end of the experimental period, considered as the final stage of fouling. A TMP jump was observed and flux was decreased rapidly in the third step. In the third step, biofilm development was observed on the membrane surface. Thus, the results suggest that the biofilm was matured by inducing a three-dimensional biofilm and the TMP reached at 41 kPa until the end of the day. On the other hand, the TMP change in the R_N_ reactor was divided into two stages (Fig. 2C). The first step of the R_N_ reactor was longer than that for the R_L_ reactor. The second step might be from the 54th day. The biofilm was observed in the final step. In conclusion, membrane fouling involving biofilm formation was developed after microbial lysis had occurred. These results indicate that fouling development is related to microbial lysis and that fouling might induce abrupt biofilm formation.

### Microbial community structure in different conditions

#### Comparison of microbial communities at a higher taxonomic level among AS, MS, and BF in each reactor

**Figure 3:**
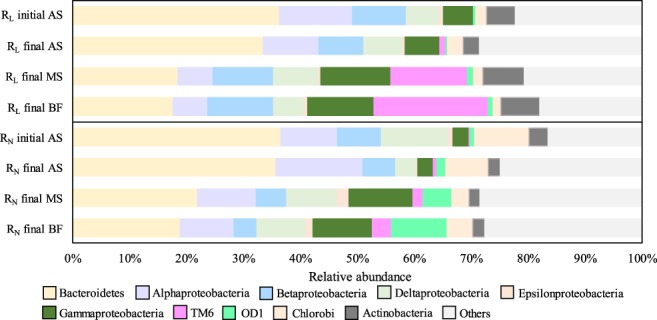
Compositional changes in the microbial community structure of the both MBRs under different conditions at the phylum or class level. AS; activated sludge, MS; membrane sludge, BF; biofilm.

Microbial samples from AS, MS, and BF were collected before and after fouling development (Fig. 2C,D). The MiSeq profile was drawn using the QIIME software to analyze the top 10 of the microbial community structure at the phylum or class level in the initial AS, final AS, MS, and BF during the operational term in each reactor (Fig. 3). Bacteroidetes, Alphaproteobacteria, Betaproteobacteria, Deltaproteobacteria, and Gammaproteobacteria was the predominant bacterial phylum or class in the AS of each reactor. Chlorobi was the predominant bacterial phylum in the AS of the R_N_ reactor. The remaining phylogenetic groups of the AS were Epsilonproteobacteria, TM6, OD1, and Actinobacteria phylum. The predominant phylum or class composition of the AS detected in each reactor was similar to the bacteria observed in the AS of the MBR treating municipal wastewater^25,26^, because this study used actual sewage as the influent.

There were clear differences between the AS and BF in each reactor, with respect to the distribution of the phylum TM6, OD1, and Gammaproteobacteria class. In the BF of the R_L_ reactor, the composition of TM6 (20.1%), Actinobacteria (6.8%) and Betaproteobacteria (11.6%), and Gammaproteobacteria (11.7%) was higher than that in the final AS. On the other hand, in the BF of the R_N_ reactor, the composition of OD1 (9.8%) and TM6 (3.3%), Deltaproteobacteria (8.6%) and Gammaproteobacteria (10.4%) was higher than that in the final AS. The microbial community structure of the cake layer was insignificantly correlated with the dominant bacteria of the mixed liquor in the MBR^27,28^. The distribution of Proteobacteria in the BF was changed from that in the final AS in each reactor. In addition, the composition of TM6 in the BF of the R_L_ reactor and TM6, OD1 in the BF of the R_N_ reactor were increased from the MS of the fouled membrane in each reactor. These results indicated that the increased bacterial phylum or class in the BF were seemed to relate with biofilm formation. In fact, Betaproteobacteria and Gammaproteobacteria are known as pioneers of fouling development^9,10^. Moreover, filamentous bacteria such as some Actinobacteria species have been reported as fouling-related bacteria^29^, which could be a reason for the increased Actinobacteria composition in the BF of the R_L_ reactor. On the other hand, the composition of Bacteroidetes was significantly decreased in the BF from the AS in each reactor. This is consistent with a previous study that reported Bacteroidetes to be decreased in the cake sludge from activated sludge^28^.

In the family level microbial community, there were also clear difference between AS and BF in each reactor (Table S1). Although families Rhodocyclaceae and Comamonadaceae commonly existed in the AS and BF samples of each reactor, family Xanthomonadaceae compositions of the BF were higher than final AS in each reactor. The family Xanthomonadaceae was reported as fouling-causing bacteria^7^. Thus, these results suggested that these bacterial groups with higher relative abundance than AS might be biofilm-forming bacteria.

#### Comparison of microbial community structure at the OTU level

**Figure 4:**
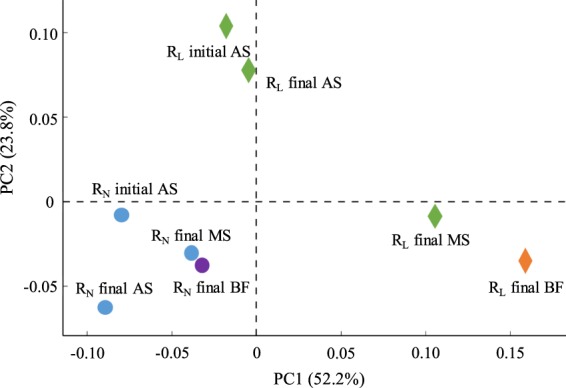
Comparison of the AS, MS and BF microbial community in both MBRs under different conditions described by a principal composition analysis (PCA) plot obtained from OTUs.

Employing MiSeq-sequencing, 742–1840 OTUs were obtained from each sample. To compare the microbial community of each sample, community profiles were visualized using a PCA plot. Figure 4 shows the PCA plot of the microbial community at the OTU level obtained from the AS, MS, and BF in each reactor. Since the distances on the plot between the initial and final AS microbial community of each reactor under the different conditions were close, no clear differences in microbial community were found between the initial and final AS of each reactor. This indicates that the microbial community structure was stable during the experimental period. In contrast, the final BF and MS plot in each reactor was differed from with each final and initial AS plot, suggesting that unique microbial communities were developed on the membrane surface as a biofilm. In addition, the microbial structure of the BF in the R_L_ and R_N_ reactors was significantly different at the OTU level. The major bacterial species involved in biofilm formation might be thus differ in each reactor.

PICRUSt analysis shows the composition difference between the BF and AS with respect to predictive functional genes related to biofilm formation and enzymes (Fig. S2). The percentage of the motility quorum-sensing regulator (MqsR) gene in the BF of R_L_ reactor was increased compared to that in the AS (Fig. S2A). In addition, the percentage of acyl homoserine lactone (AHL) synthase, which generates a kind of auto-inducer molecules, was increased in the BF of each reactor compared to the AS (Fig. S2B). MqsR is correlated with an increase in biofilm formation^30^ and AHL is also reported to correlate with biofilm formation and bacterial growth^31^. Thus, these findings suggest that the unique microbial community developed on the membrane surface might affect the function of biofilm communities. In conclusion, the difference in microbial communities between the BF and AS was influenced by unique bacteria such as the biofilm-forming bacteria in each reactor.

#### Biofilm-forming bacteria in both reactors under different conditions

The top 10 ranked OTUs of BF samples are shown in Table 2, and were selected based on increasing ratios based on the final AS in each reactor (Fig. S3). In the BF of the R_N_ reactor, the most dominant OTU (denovo3418) was *Dokdonella* sp., which showed a high increasing ratio in the final AS and possesses lipase activities^32^. In previous studies on biofilms or granular sludge, these bioaggregates were considered to comprise proteins, polysaccharides, lipids and microbial cells^33,34^. In fact, some predictive lipase percentage in the BF was higher compared to the final AS in both reactors in this study (Table S2). These results imply that the biofilm maturation was facilitated by the presence of particular bacteria, which possess enzymatic activities such as lipases and proteases, and formed lower molecules present in biofilms, such as SMPs. The OTUs assigned to the uncultured bacterial phyla TM6 (denovo6461), OD1(denovo5772, denovo6080), and GN02 (denovo798) were subsequently predominant in the R_N_ reactor. OD1 was detected in the biofilm on the membrane surface of fouled MBR and might be related to biofilm formation^7,35^. The remaining OTUs of normal BF were uncultured *Myxococcales* (denovo3208) and *Polyangium* (denovo6607) belonging to order *Myxococcales* and myxobacteria have been reported to produce colloid to form biofilm and cause fouling^36^. The *Saprospira* sp. (denovo6316) is related to cell lysis^37^, and thus, its presence might facilitate the assimilation of microbes in the biofilm.

**Table 2:** The top 10 increased OTUs in BF compared with the final AS of each reactor*.

OTU ID	Phylogenetic affiliation	Relative abundance
Normal BF (R_N_)		
denovo6461	Candidate division TM6 phylum	2.439%
denovo3418	*Dokdonella* sp. (class Gammaproteobacteria)	5.405%
denovo5772	Candidate division OD1 phylum	1.932%
denovo798	Candidate division GN02 phylum	0.880%
denovo3208	Uncultured Myxococcales (class Deltaproteobacteria)	0.928%
denovo6607	*Polyangium* sp. (class Deltaproteobacteria)	0.794%
denovo6080	Candidate division OD1 phylum	0.784%
denovo5106	Uncultured Deltaproteobacteria	0.928%
denovo658	*Desulfatiglans* sp. (class Deltaproteobacteria)	0.689%
denovo6316	*Saprospira* sp. (phylum Bacteroidetes)	0.555%
Low OLR BF (R_L_)		
denovo6461	Candidate division TM6 phylum	19.414%
denovo5106	Uncultured Deltaproteobacteria	2.604%
denovo5366	Unclassified Neisseriaceae (class Betaproteobacteria)	3.478%
denovo6762	*Conexibacter* sp. (phylum Actinobacteria)	2.113%
denovo2742	Unclassified Neisseriaceae (class Betaproteobacteria)	1.038%
denovo6851	Unclassified Rubrobacteria (phylum Actinobacteria)	1.329%
denovo3909	*Legionella* sp. (class Gammaproteobacteria)	0.692%
denovo516	*Rudaea* sp. (class Gammaproteobacteria)	1.512%
denovo4166	Unclassified Neisseriaceae (class Betaproteobacteria)	0.874%
denovo1332	*Legionella* sp. (class Gammaproteobacteria)	0.656%

*The ratio of the increase was calculated using STAMP software and the results are presented in Supplementary Fig. S3.

Conversely, in the BF of the R_L_ reactor, OTU assigned to the candidate phylum TM6 (denovo6461) was the most dominant. McLean *et al*.^38^ reported that TM6 bacteria were detected in a biofilm from a sink drain in a hospital restroom. In addition, the previous study suggested that TM6 was the predominant bacteria in an anaerobic MBR reactor^39^. These reports indicate that TM6 might survive in an anoxic or anaerobic environment. Biofilms form a partial anoxic zone located between the membrane surface and the membrane sludge cake, which could be a reason for the increased TM6 composition. Unclassified *Neisseriaceae* (denovo5366, 2742, 4166: total detection rate; 5.390%) belonging to Betaproteobacteria ranked next in predominance. Betaproteobacteria are also reported to play an important role in mature biofilm formation in MBRs^9^. *Conexibacter* (denovo6762: 2.113%) which was detected in biocathode biofilms^40^, might be related to biofilm formation. In addition, *Legionella* sp. (denovo3909, 1332: total 1.348%) was present in a protozoan host and survived within a biofilm matrix^41,42^. The difference of predominant OTUs in the BF of each reactor might depend on the operational condition of the A/O-MBR, but some similar bacterial groups were observed.

The top 5 shared OTUs in BF samples from both R_N_ and R_L_ reactors that showed increased detection ratio compared to that in the final AS are shown in Table 3. Both BFs showed a higher detection ratio for OTUs classified as uncultured bacterial groups of the candidate phylum TM6 (denovo6461), uncultured Deltaproteobacteria (denovo5106), and uncultured *Myxococcales* (denovo3208). Interestingly, among the top 5 most abundant OTUs in both BF samples, 4 OTUs from the BF of R_L_ reactor showed a higher abundance rate than that of the BF of R_N_ reactor. This result suggested that the low OLR condition could promote biofilm formation, which is similar microbial compositions of normally formed biofilm.

**Table 3:** The top 5 most abundant shared OTUs in BF samples between the RN and RL reactors selected as increasing bacteria compared with the final AS in each reactor*.

OTU ID	Phylogenetic affiliation	Relative abundance	
		Normal BF (R_N_)	Low OLR BF (R_L_)
denovo6461	Candidate division TM6 phylum	2.439%	19.414%
denovo5106	Uncultured Deltaproteobacteria	0.928%	2.604%
denovo3208	Uncultured Myxococcales (class Deltaproteobacteria)	0.928%	0.310%
denovo643	*Thalassolituus* (class Gammaproteobacteria)	0.41%	0.42%
denovo4372	*Fischerella* (phylum Cyanobacteria)	0.29%	0.53%

*The ratio of the increase was calculated using STAMP software and the results are presented in Supplementary Fig. S3.

A TMP jump is induced by the existence of an anoxic zone in the interior of a biofilm^43,44^. A previous study reported that bacteria belonging to TM6, *Desulfatiglans* and *Rudaea* thrived under anaerobic or oxic conditions^39,45,46^. We considered this was the reason for the high abundance of TM6, *Desulfatiglans* and *Rudaea* (Tables 2, 3). Our findings show that various microorganisms such as biofilm forming bacteria, which mainly include uncultured bacteria, biofilm-utilizing bacteria, and the partner, were present in both biofilms. However, the relationship between temporal bacterial growth and biofilm formation is still unclear. Thus, the bacterial species involved in biofilm formation and TMP behavior should be investigated simultaneously in future studies. In addition, biofilms show complex interactions among bacterial microorganisms as well as eukaryotic microorganisms^47^. Thus, an investigation of the microorganism network including the metazoans and protozoans in biofilms is required.

## Conclusion

In the A/O-MBR operated under low organic loading rate condition (R_L_ reactor; OLR: 0.002 kg-COD·m^−3^·day^−1^), membrane fouling and biofilm were developed rapidly compared to the A/O-MBR under normal conditions (R_N_ reactor; OLR: 0.42 kg-COD·m^−3^·day^−1^). The microbial community composition between the bulk AS and BF was considerably different, and characteristic bacteria found in BF were thought to important for biofilm formation on the membrane surface in A/O-MBR. TM6 showed specific presence on the fouled membrane surface as a biofilm in the R_L_ reactor. On the other hand, OD1 was the predominant phylum in the fouled membrane surface of the R_N_ reactor. In addition, biofilms might be formed by the same process in both reactors. However, correlation of the bacterial species involved in biofilm formation and TMP behavior should be investigated in future studies.

## Electronic supplementary material

Supplementary Information
