# Supplementary material for: Fouling Development in A/O-MBR under Low Organic Loading Condition and Identification of Key Bacteria for Biofilm Formations
Source: Sci Rep. 2018 Jul 30;8:11427. doi: 10.1038/s41598-018-29821-9 (PMC6065318; doi:10.1038/s41598-018-29821-9)
Supplement: Supplementary file 1 — Supplementary Information [file 41598_2018_29821_MOESM1_ESM.pdf]

Supplemental Information

Fouling Development in A/O-MBR under Low Organic Loading Condition and Identification of Key Bacteria for Biofilm Formations

Yuya Takimoto[1], Masashi Hatamoto[2,3*], Takaya Ishida[2], Takahiro Watari[2], Takashi Yamaguchi[1,2]

[1]Department of Science of Technology Innovation, Nagaoka University of Technology, 1603-1 Kamitomioka, Nagaoka, Niigata 940-2188, Japan

[2]Department of Civil and Environmental Engineering, Nagaoka University of Technology, 1603-1 Kamitomioka, Nagaoka, Niigata 940-2188, Japan

[3]Top Runner Incubation Center for Academia-Industry Fusion, Nagaoka University of Technology, 1603-1 Kamitomioka, Nagaoka, Niigata 940-2188, Japan

*Corresponding author

Department of Civil and Environmental Engineering, Nagaoka University of Technology, 1603-1 Kamitomioka, Nagaoka, Niigata 940-2188, Japan

Tel : +81-258-47-9637 ; Fax : +81-258-47-9637

E-mail address : hatamoto@vos.nagaokaut.ac.jp (Masashi Hatamoto)

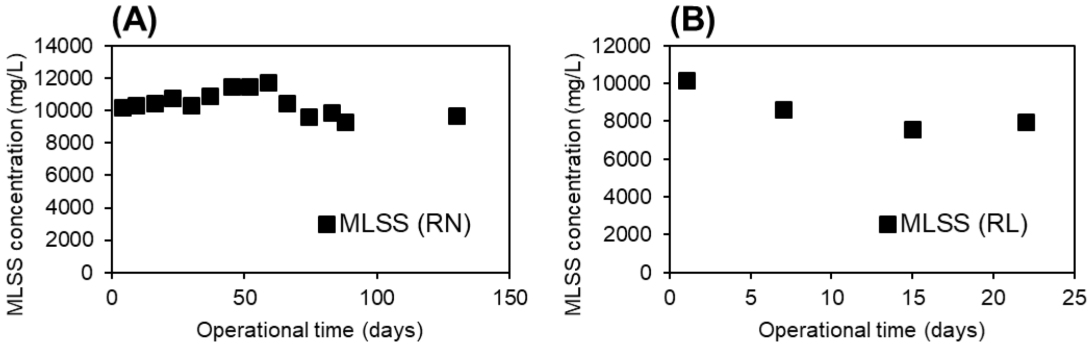

Figure S1. Changes in the MLSS concentration of the aerobic tank. (A): R_N reactor, (B): R_L reactor.

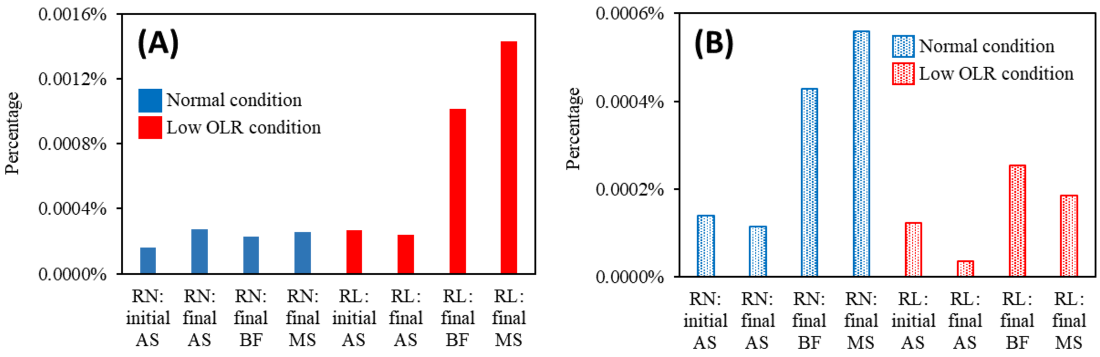

Figure S2. Prediction of functional genes according to PICRUSt analysis of the initial and final AS, final MS and BF in each reactor. (A) a motility quorum-sensing regulator gene, (B) an acyl homoserine lactone synthase (auto-inducer) gene.

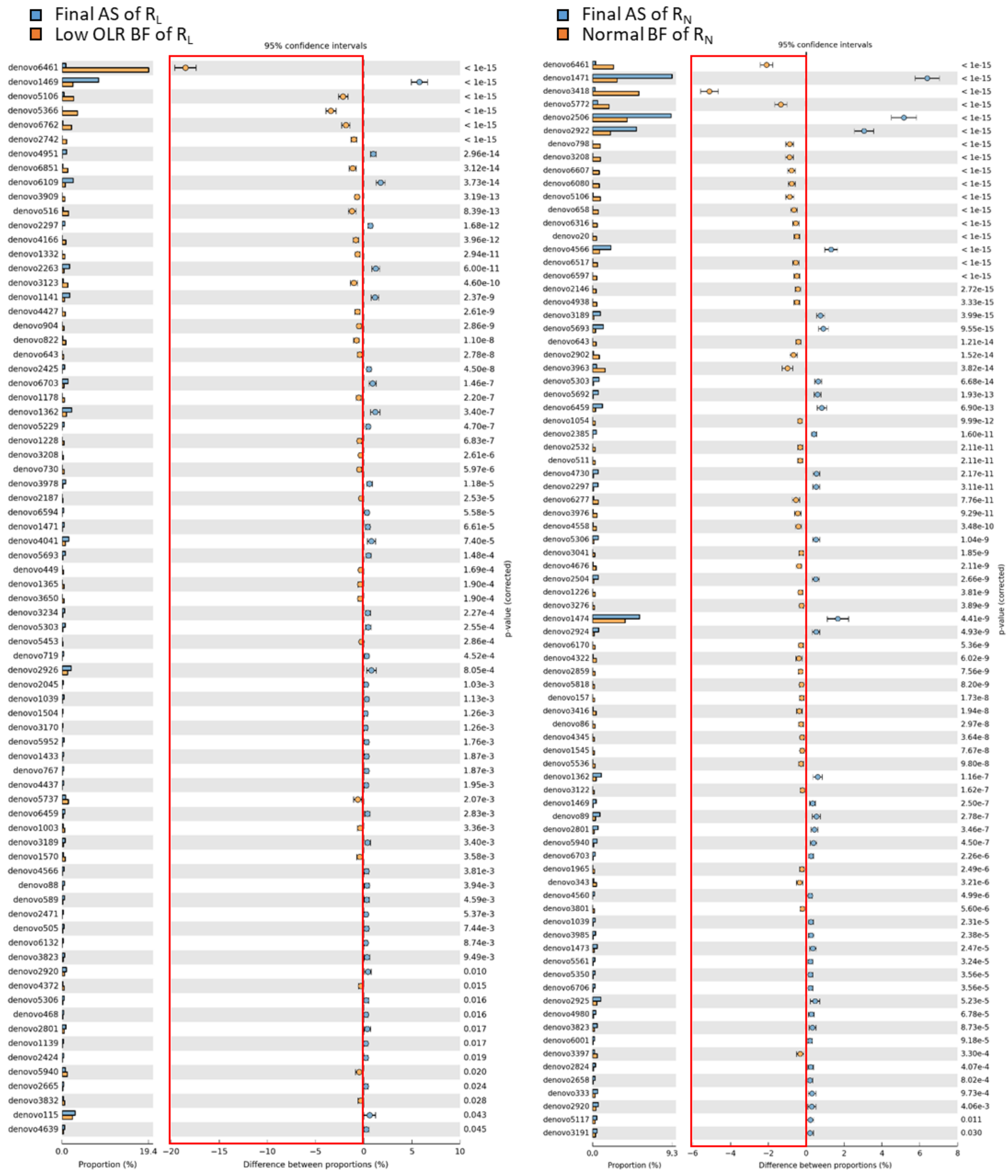

Figure S3. Increased OTUs of BF compared with the final AS in each reactor by STAMP software.

Table S1. The top 30 ranked most abundant in the

No.	Phylogenetic affiliation	Relative abundance							
		R_L initial AS	R_L final AS	R_L final MS	R_L final BF	R_N initial AS	R_N final AS	R_N final MS	R_N final BF
1	Phylum Bacteroidetes: Family Saprospiraceae	6%	4%	2%	2%	18%	23%	10%	9%
2	Phylum TM6; Class Unclassified SJA-4	0%	1%	13%	20%	0%	0%	1%	3%
3	Phylum Bacteroidetes; Family Chitinophagaceae	17%	16%	8%	7%	8%	5%	3%	3%
4	Class Unclassified Alphaproteobacteria	0%	0%	0%	0%	1%	10%	6%	5%
5	Phylum Bacteroidetes; Order Unclassified Sphingobacteriales	8%	7%	4%	5%	5%	5%	4%	4%
6	Class Deltaproteobacteria; Order Unclassified Myxococcales	2%	3%	1%	1%	8%	1%	3%	2%
7	Class Betaproteobacteria; Family Procabacteriaceae	0%	0%	6%	7%	0%	0%	1%	0%
8	Class Gammaproteobacteria; Family Xanthomonadaceae	2%	3%	5%	5%	1%	1%	7%	7%
9	Phylum Bacteroidetes; Family Cryomorphaceae	0%	0%	0%	0%	1%	1%	2%	1%
10	Phylum Chlorobi; Class Unclassified SJA-28	0%	1%	0%	0%	4%	6%	2%	4%
11	Class Betaproteobacteria; Family Rhodocyclaceae	3%	3%	2%	2%	4%	3%	2%	1%
12	Phylum Chlorobi; Class Unclassified OPB56	1%	2%	1%	1%	5%	1%	1%	0%
13	Class Alphaproteobacteria; Family Sphingomonadaceae	3%	2%	1%	1%	1%	1%	0%	1%
14	Class Unclassified Deltaproteobacteria	0%	1%	4%	3%	0%	0%	3%	3%
15	Class Betaproteobacteria; Family Comamonadaceae	3%	2%	2%	2%	3%	2%	1%	1%
16	Phylum OD1; Class Unclassified ZB2	0%	0%	1%	0%	0%	0%	2%	4%
17	Phylum Actinobacteria; Order Unclassified Acidimicrobiales	3%	1%	1%	1%	2%	1%	0%	0%
18	Class Alphaproteobacteria; Famliy Methylocystaceae	1%	1%	0%	0%	3%	1%	0%	0%
19	Phylum OD1; Class Unclassified ABY1	0%	0%	0%	0%	0%	1%	2%	3%
20	Phylum Bacteroidetes; Order Unclassified Saprospirales	3%	3%	1%	2%	1%	1%	0%	0%
21	Class Gammaproteobacteria; Family Legionellaceae	0%	0%	3%	2%	0%	0%	1%	1%
22	Phylum Nitrospirae; Family Nitrospiraceae	2%	2%	1%	1%	2%	2%	1%	2%
23	Phylum Actinobacteria; Family Patulibacteraceae	0%	0%	3%	2%	1%	0%	0%	0%

24	Class Alphaproteobacteria; Family Hyphomicrobiaceae	2%	1%	1%	1%	1%	1%	0%	1%
25	Phylum Bacteroidetes; Family Cytophagaceae	1%	2%	1%	1%	1%	1%	0%	1%
26	Class Betaproteobacteria; Family Oxalobacteraceae	1%	1%	1%	1%	0%	0%	1%	0%
27	Phylum Planctomycetes; Order Unclassified agg27	1%	2%	1%	1%	0%	0%	0%	0%
28	Phylum Chlamydiae; Family Parachlamydiaceae	0%	0%	0%	0%	0%	0%	2%	2%
29	Class Alphaproteobacteria; Order Unclassified Rhizobiales	1%	2%	1%	1%	2%	1%	0%	0%
30	Class Deltaproteobacteria; Family Polyangiaceae	1%	1%	0%	0%	1%	1%	0%	1%
31	Others	36%	38%	35%	32%	27%	33%	44%	42%

Table S2. The predictive lipase percentages in AS or BF samples according to PICRUSt analysis.

Sample name	Initial AS	Final AS	Final BF	Final MS
R_N reactor				
phospholipase A1 [EC:3.1.1.32]	0.0073%	0.0059%	0.0131%	0.0148%
outer membrane lipase/esterase	0.0006%	0.0005%	0.0008%	0.0011%
phospholipase D [EC:3.1.4.4]	0.0002%	0.0001%	0.0004%	0.0003%
R_L reactor				
phospholipase A1 [EC:3.1.1.32]	0.0084%	0.0078%	0.0162%	0.0154%
outer membrane lipase/esterase	0.0012%	0.0024%	0.0060%	0.0059%
phospholipase D [EC:3.1.4.4]	0.0002%	0.0002%	0.0006%	0.0006%
